# Sin1-mediated mTOR signaling in cell growth, metabolism and immune response

**DOI:** 10.1093/nsr/nwz171

**Published:** 2019-11-19

**Authors:** Chun Ruan, Xinxing Ouyang, Hongzhi Liu, Song Li, Jingsi Jin, Weiyi Tang, Yu Xia, Bing Su

**Affiliations:** 1 Shanghai Institute of Immunology, Department of Immunology and Microbiology, and the Minister of Education Key Laboratory of Cell Death and Differentiation, Shanghai Jiao Tong University School of Medicine, Shanghai 200025, China; 2 Zhiyuan College, Shanghai Jiao Tong University, Shanghai 200240, China

**Keywords:** Sin1, AGC kinases, mTOR complex, Akt, metabolism and immune response

## Abstract

The mammalian target of rapamycin (mTOR) is an evolutionarily conserved Ser/Thr protein kinase with essential cellular function via processing various extracellular and intracellular inputs. Two distinct multi-protein mTOR complexes (mTORC), mTORC1 and mTORC2, have been identified and well characterized in eukaryotic cells from yeast to human. Sin1, which stands for *S*ty1/Spc1-*in*teracting *p*rotein1, also known as mitogen-activated protein kinase (*MAPK*) *a*ssociated *p*rotein (MAPKAP)1, is an evolutionarily conserved adaptor protein. Mammalian Sin1 interacts with many cellular proteins, but it has been widely studied as an essential component of mTORC2, and it is crucial not only for the assembly of mTORC2 but also for the regulation of its substrate specificity. In this review, we summarize our current knowledge of the structure and functions of Sin1, focusing specifically on its protein interaction network and its roles in the mTOR pathway that could account for various cellular functions of mTOR in growth, metabolism, immunity and cancer.

## INTRODUCTION

The mammalian target of rapamycin (mTOR) regulates diverse cellular and molecular functions that ultimately control cell and body growth [1–4]. Our understanding of mTOR function and regulation begins with the finding of a bacterially produced compound called rapamycin. Rapamycin was originally identified from *Streptomyces hygroscopicus* isolated from soil samples from Easter Island, known to locals as *Rapa nui* [5–7]. Rapamycin can bind to a conserved cellular protein called FKBP12 and allosterically inhibit mTOR activity [3,8,9]. The molecular function of mTOR involves the regulation of translation initiation, ribosome biogenesis, protein maturation, autophagy, actin cytoskeleton reorganization and transcription etc. [3,10–12]. Studies, using gene-deficient mice, that impair functions of mTOR and its regulators, have greatly expanded our knowledge about mTORC’s function in the development and homeostasis of different cells in different organisms [2,13–16]. On the other hand, abnormality in the mTOR-regulated pathways is associated with numerous pathological conditions including metabolic diseases, cancer, immune disorders, and cardiovascular and neurological diseases [2,17–20]. Since the mTOR pathway is extensively reviewed elsewhere [1,3,21], this review will briefly discuss the overall mTOR signaling and mainly focus on Sin1, one of the key adaptor molecules in mTORC2, for its regulation and function in the mTOR signaling pathway.

**Figure 1.**
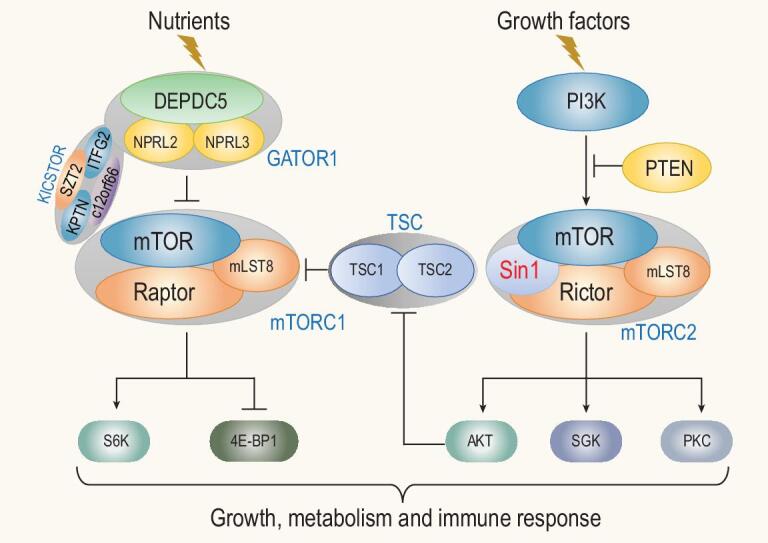
Illustration of the key components of two mTOR complexes and their responses to nutrients and growth factors. In response to nutrients and growth factors, mTORC1 and mTORC2 phosphorylate their respective substrates as indicated to control cell growth, metabolism and immunity.

Mammalian TOR belongs to a family of phosphoinositide 3-kinase (PI3K)-related kinases (PIKKs) [22,23]. Interestingly, members of this family share homology with lipid kinases but instead of lipid, they phosphorylate Ser/Thr residues in proteins [23,24]. Many PIKKs form multi-protein complexes and the binding partners often dictate their substrate specificity [25,26]. Components of the TOR pathway are generally highly conserved [3,10,27] and mTOR forms at least two distinct protein complexes termed mTOR complex (mTORC) 1 and mTORC2 (Fig. 1) that perform different cellular functions [2,10]. mTORC1 is sensitive to rapamycin and nutrient status [12,28]. In contrast, mTORC2 only responds partially to rapamycin treatment and is stimulated by growth factors [10,29].

mTORC1 core components consist of mTOR, regulatory-associated protein of mTOR (Raptor) and mLST8 (also called GβL) [28,30,31]. Other subunits include PRAS40 and Deptor [32,33] (Fig. 1). mTORC1 responds to nutrients, for example amino acids and glucose. These signals can regulate mTORC1 temporally and spatially via phosphorylation of Raptor [34]. mLST8, a protein consisting of seven WD40 repeats, is an essential subunit not only for mTORC1 but also mTORC2. Consistently, mLST8 ablation in mice completely abolishes mTORC2 activity [35]. mLST8 binds to the kinase domain of mTOR to stimulate mTOR catalytic activity [36]. Recent structural study of the mTOR complex has revealed parts of the mTORC1 structure at the atomic level [37], confirming such an mLST8–mTOR interaction. mTORC1 additionally interacts with PRAS40, FKBP38 and Deptor to control the activity of mTORC1 under different growth conditions [27,38].

The activity of mTORC1 is controlled by diverse cues ranging from the levels of amino acids, glucose, oxygen, energy and redox status, to mitogens such as growth factors and cytokines, and is acutely inhibited by rapamycin [39–41]. When amino acids are sufficient, small GTPases Rag (heterodimers of either RagA or RagB with either RagC or RagD) becomes active, which in turn anchors mTORC1 to lysosomes [40,42]. Cellular redox status also regulates mTORC1 activity via cysteine oxidants in the absence of amino acids [39,43]. In contrast, growth factors induce mTORC1 activation via the PI3K–Akt signaling pathway [44]. Activated Akt is able to inactivate the tuberous sclerosis complex (TSC1/TSC2) proteins by phosphorylation of TSC2, which is a GTPase-activating protein (GAP) for Rheb, a positive regulator of mTORC1, thus leading to augmented mTORC1 activity [45]. Multiple pathways in mammals convey the growth and nutritional signals to mTORC1 via regulation of TSC1/TSC2 [46] (Fig. 1).

mTORC2 contains the conserved core components rapamycin-insensitive component of TOR (Rictor), Sin1 and mLST8, and other proteins including PRR5/Protor and PRR5L [47–49]. The stability and integrity of mTORC2 depend on both Rictor and Sin1. Rictor has a N-armadillo domain and a conserved C-terminus that contains multiple potential phosphorylation sites [50]. Among these sites, Thr1135 is the target of AGC family kinases such as S6K and Akt, and thus regulated by various nutrients and growth factors [51,52]. Interestingly, Thr1135 phosphorylation seems not to affect the phosphorylation of the well-known mTORC2 substrates Akt and SGK1. Instead, it may disrupt the association of Rictor and Cullin-1, affecting the ubiquitination of SGK1 [52]. Sin1 deficiency causes a decreased protein level of Rictor and abolishes Rictor interaction with mTOR [10,53], while Rictor-deficiency also affects Sin1 protein level [35,53,54], suggesting that they may require each other for stability.

Besides the well-studied mTORC1 and mTORC2 complexes, recent proteomic studies have identified various proteins associated with key subunits of mTOR such as Sin1 and mLST8 [55]. They form mTOR-like complexes and regulate important cellular processes under specific physiological conditions. For example, in lymphoma cells, CDK9 interacts with mLST8 in nuclei and promotes transcription of leukemogenesis-related genes [55]. CDK9 may also interact with Sin1/Rictor in mTORC2 for LARP1 phosphorylation to potentially promote tumor growth [55]. Deletion of Rictor in mouse brown adipocytes led to increased lipid intake and catabolism, which appeared to be independent of Akt, suggesting a non-canonical mTOR signaling pathway [55]. There was an increased mTOR/SIRT6 interaction and FoxO1 deacetylation in Rictor-deficient brown adipocytes, which may be the cause of increased lipid intake and catabolism [56]. However, in the following sections, we will review in detail the structure and function of Sin1, and discuss recent development of Sin1-mediated mTOR signaling in cell growth, metabolism and immune function.

## SIN1 IS A CONSERVED ADAPTOR MOLECULE ESSENTIAL FOR mTORC2 FUNCTION

### Discovery of Sin1

*S*ty1/Spc1-*in*teracting protein 1 (Sin1), also known as mitogen-activated protein kinase (*MAPK*) *a*ssociated *p*rotein (MAPKAP)1, was first identified in 1999 in fission yeast *Schizosaccharomyces pombe* as an adaptor protein interacting with Sty1 (also known as Spc1), a member of the eukaryotic stress-activated protein kinase (SAPK) or mitogen-activated protein kinase (MAPK) family [57]. Under cellular stresses, Sin1 is phosphorylated in a Sty1/Spc1-dependent manner. Interestingly, Sin1 is not required for Sty1/Spc1 activation but is required for stress-dependent transcription via its substrate, Atf1. Cells lacking Sin1 display multiple stress sensitivity including heat shock, osmotic stress, oxidative stress and so on [58]. In mammalian cells, Sin1 was also found to negatively regulate the MAPK pathway via direct interaction with MAP3K2, MEKK2 [59]. Later through both biochemistry and genetics studies, Sin1 was shown to be essential for the assembly and substrate specification of mTORC2 [10,53]. Intensive studies of mammalian Sin1 in mice and cell lines have revealed the essential roles of Sin1 in embryonic development, cancer, immune function and other organ development that will be discussed in more detail in the following sections.

### Conservation of the Sin1 molecule

**Figure 2.**
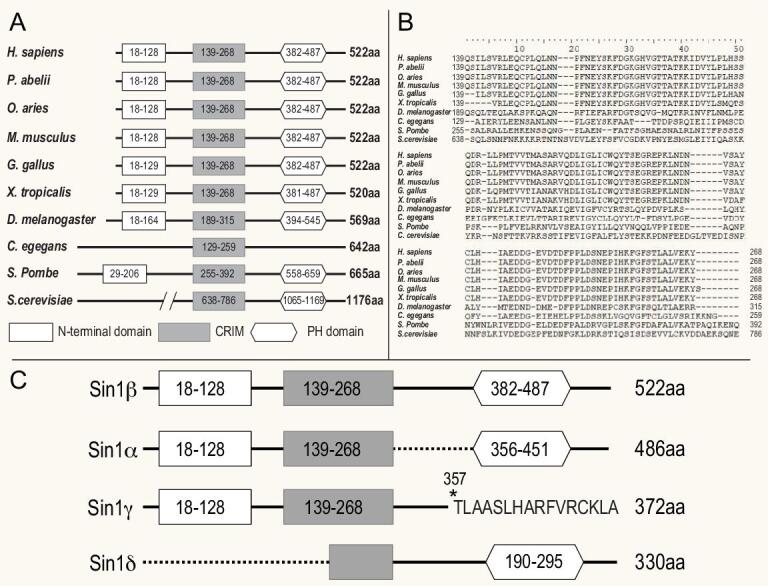
Sequence alignment of evolutionarily conserved Sin1 proteins. (A) A diagram showing the evolutionarily conserved domains in Sin1 proteins among different species. Empty rectangle, N-terminal region; filled rectangle, conserved region in the middle (CRIM); hexagon, PH domain. (B) Sequence alignment of the CRIM domain of Sin1 from different species. (C) Alternative splicing isoforms of human Sin1.

The Sin1 protein is conserved from yeast to mammals and its orthologues are highly conserved in vertebrates from fish to mammals [59–62] (Fig. 2A). This high conservation of Sin1 suggests that Sin1 may play an indispensable role in eukaryotic organisms. However, Sin1 orthologue is not found in Plantae genomes [63]. Comparison of the conserved sequences in various Sin1 orthologues has identified a stretch of sequence called conserved region in the middle (CRIM) that is conserved among all species including *Saccharomyces cerevisiae* [64] (Fig. 2B). In addition, a putative PH domain at the C-terminus is also conserved in most species except the worm *Caenorhabditis elegans*. Interestingly, the N-terminal region with about 100 amino acids of Sin1, which is sufficient for the assembly of mammalian TORC2 [36] (unpublished data), is missing in the worm and *S. cerevisiae* (Fig. 2A) [63]*.* Together, these data demonstrate that Sin1 structure and function are well conserved in various species (data based on Uniprot sequence).

An adaptor protein called Avo1 in the budding yeast *S. cerevisiae* was first studied as the key component of *S. cerevisiae* TORC2, which also contains TOR2, AVO2 (Rictor orthologue) and AVO3 (mLST8 orthologue) [31,65]. However, it was not considered as a Sin1 orthologue due to its low sequence homology (about 20%) with other Sin1 orthologues, and because of this, mammalian Sin1 was first thought not to be involved in mTOR function [1,31]. The Avo1-containing [65] TORC2 in *S. cerevisiae* was shown to regulate actin organization and maintain cell wall integrity via Ypk2 (yeast protein kinase 2) [66]. Interestingly, the conserved CRIM domain in Avo1 was found to recruit Ypk to TORC2 [31,67]. In addition to the CRIM domain, Avo1 also contains a putative PH domain (840–933 amino acids) and a sequence with weak homology to Ras-binding domain (RBD) found in many Ras target proteins [31] (Fig. 2A). This RBD domain is also found in the *Dictyostelium discoideum* Sin1 orthologue RIP3, indicating that Sin1 in these two species may bind and inhibit Ras protein signaling [68]. Such RBD sequence is also identified in mammalian Sin1, strongly suggesting that Sin1 plays an important role in regulating Ras signaling [10,58,59,69,70].

### Mammalian Sin1 structure

Like many other members of the Sin1 family, mammalian Sin1 protein contains an N-terminal region, a CRIM domain (conserved region in the middle), a RBD [68] (Ras-binding domain) and a PH (pleckstrin homology) domain in its C-terminal region (Fig. 2A). The N-terminal region of mammalian Sin1 is important for interaction with either Rictor or MEKK2 [36,59]. The RBD motif in mammalian Sin1 is suggested to bind to Ras protein and inhibit the Ras signaling [68]. The CRIM domain of Sin1 is highly conserved among all Sin1 orthologues [64]. It likely serves as a specific binding site for many of the mTORC2 substrates and thus contributes to the specificity of mTORC2 [71,72].

The PH domain in the C-terminal region of Sin1 contains a lipid-binding motif that could selectively bind phosphor-inositides in cellular membrane [71]. In PI3K-mediated mTORC2 activation, the PH domain of Sin1 specifically interacts with phosphatidylinositol (3,4,5)-trisphosphate [73] on the plasma membrane and is involved in mTORC2 activation via a ‘release-of-inhibition’ mechanism [72]. The PH domain is not required for the enzymatic activity of mTORC2 *in vitro* since the kinase activity of mTORC2 is comparable between full-length and PH domain-truncated Sin1 [36]. The Sin1 PH domain was also shown to interact with PKC [74,75], suggesting that Sin1 may regulate the activity of other kinases using similar protein–lipid interaction [72,76].

Detailed Sin1 structure studies using NMR, crystallization and cryo-EM have shed light on its function as well as evolution. The crystal structures of yeast and human Sin1 PH domains have revealed that though they adopted similar PH folding, their binding pockets for phospholipids are not the same, indicating that during evolution, Sin1 may evolve to recognize different substrates [77]. An NMR study of Sin1 CRIM domain in fission yeast *S. pombe* showed that it formed a ubiquitin-like structure with a characteristic acidic protrusion which contributed to the recruitment of TORC2 substrate Akt. Notably, this yeast Sin1 CRIM domain can be fused to any other subunits of TORC2 and retains the complex's kinase activity, even in Sin1-null cells [71]. In contrast, the Sin1 CRIM domain in budding yeast and human is not sufficient for the integrity and function of mTORC2 [36,78]. Recent cryo-EM studies have shed light on Sin1 structure in the context of the TORC2 protein complex. The yeast TORC2 contains two copies of Sin1 (Avo1) and it functions as a scaffold for the complex by interacting with almost all other subunits, including LST8, Avo3 and TOR [78]. In human mTORC2, mammalian Sin1 interacts with Rictor to form a steric hindrance near the rapamycin–FKBP12 complex binding region in mTOR. This may explain why mTORC2 is insensitive to rapamycin inhibition. However, due to the flexibility of Sin1 protein, its accurate conformation in the mTORC2 complex remains elusive in all structural studies so far [36].

### Sin1 isoforms and intracellular localization

Mammalian Sin1 exists in multiple alternatively spliced isoforms (Fig. 2C) [60,64,79]. At least five different transcript variants and four distinct protein isoforms have been identified [54,64,80]. These isoforms vary in expression level, and cellular and tissue location [54,80]. Sin1α and Sin1β are the two main isoforms that form mTORC2. Sin1γ, which lacks the PH domain, cannot be localized to the plasma membrane. Instead, it was found to be co-localized with the centrosome and forms a distinct cylinder structure, and may regulate cell division [80]. Notably, Sin1γ-containing mTORC2 is resistant to insulin stimulation and its enzymatic activity is dramatically reduced [54,80]. Sin1δ, which lacks an N-terminal domain, does not co-immune precipitate with other components of mTORC2 and is not responsible for most of the mTORC2 functions, such as phosphorylation of Akt at Ser473 and the formation of a Sin1–Rictor–mTOR complex [80]. Its precise function remains unclear. These observations confirm the importance of the N-terminal region of Sin1 for mTORC2 complex integrity.

Subcellularly, active mTORC1 mainly localizes on the surface of lysosomes [40] while mTORC2 was originally reported to be present on mitochondria-associated endoplasmic reticulum membranes [81]. Using a cell-compartment-specific reporter system, Ebner *et al.* broadened the localization of mammalian Sin1 to many organelles such as plasma membrane, mitochondria outer membrane and endosomes. Notably, the subcellular localization of mTORC2 affects its response to PI3K signaling [82]. Extracellular signals such as hormones also regulate mTORC2 function by dictating its subcellular localization [83]. Another example is upon activation of iNKT cells, augmented glycolysis activates mTORC2 by recruiting it to mitochondria via hexokinase-II. This step is crucial to regulate the production of IFN-γ in matured iNKT cells [82,84]. However, how, and the precise cellular location by which mTORC2 is regulated in catalyzing the turn motif phosphorylation of Akt and cPKC remain unclear [85,86].

### Sin1 interacts with many protein kinases

Early studies of mouse and human Sin1 show that it is an interacting protein for MAP3K2 MEKK2 and involved in negatively regulating the MAPK signaling [59]. Sin1 could form a stable complex with the inactive and non-phosphorylated MEKK2, thereby preventing its dimerization and activation, resulting in inhibition of MEKK2/c-Jun N-terminal kinase (JNK) signaling [59,79]. Another study showed that human Sin1 and its isoform Sin1α bound to JNK *in vitro* and *in vivo*, but not to p38- or ERK1/2-family MAPKs [61]. Overexpression of full-length Sin1 suppressed basal JNK activity and UV-C-induced activation of JNK in certain cell types, suggesting that Sin1 may serve as a scaffold protein in the regulation of JNK signaling [61]. In another study, it was shown that Sin1 was capable of binding to both ATF-2 and p38, and enhanced ATF-2-dependent transcription in an SAPK signaling pathway [87]. It was also reported that ovine Sin1 (ovSin1) could bind with ovIFNAR2 constitutively and the two proteins were co-localized to the plasma membrane and perinuclear structures [88]. Furthermore, it was reported that Sin1 may have specific and conserved sequences for IFNAR2 interaction [60]. These studies may provide a possible link between type I IFN function and Sin1-mediated signaling pathways. In this regard, it was reported that Sin1/mTORC2 played a role in type I IFN-induced expression of ISG and type I IFN biological responses via engagement of the Akt/mTORC1 axis, including IFN-induced phosphorylation of S6 kinase and phosphorylation of 4EBP1 [89]. In addition, targeted disruption of Sin1 led to decreased activation of the STAT1 signaling pathway and type I IFN-induced gene transcription in antiproliferative responses [90]. More recently, it was found that Sin1 could regulate IFNγ-induced genes and type II IFN-mediated biological responses via both Akt/mTORC1 activation and tyrosine phosphorylation of STAT1 [91].

### Sin1 is essential for Akt HM and TM site phosphorylation

The most characterized mTORC2 substrates include Akt, conventional PKC, and other AGC (protein kinase A, G, C families) protein family members [4]. Although studies using Sin1-deficient cells first confirmed the essential role of Sin1 in mTORC2-mediated phosphorylation of Akt Ser473 at its hydrophobic motif [10,53], it did reveal another important function of Sin1 and mTORC2 until later. It was known for quite a long time that Akt is constitutively phosphorylated at the Thr450 (murine, human Thr451) site within its turn motif [92,93]. Due to its constitutive nature, this phosphorylation was first thought to have no significant function [92]. It was first speculated to be an autophosphorylation. Later studies suggest that it may contribute to the induction of Akt activity [94]. However, following the identification of Sin1 as the most important component of mTORC2 and required for the assembly of the kinase complex for Akt Ser473 phosphorylation, it was soon shown that Sin1-mTORC2 also served as the kinase for Akt Thr450 and PCK at the turn motif (TM) independent of Ser473 phosphorylation [85,86].

**Figure 3.**
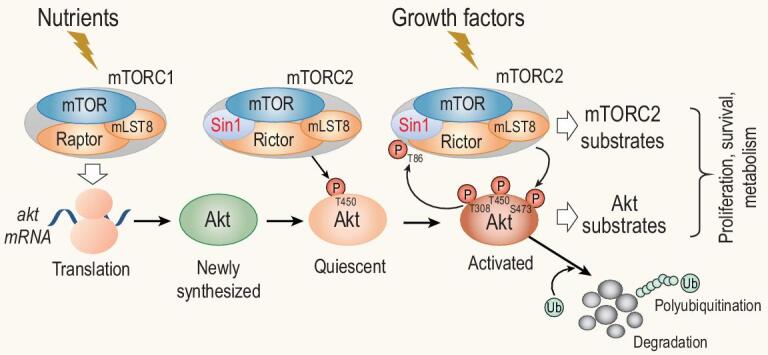
mTORC1 and mTORC2 regulate Akt protein synthesis, stability and activation. When nutrients are sufficient, mTORC1 is activated to direct Akt protein synthesis. During Akt translation, mTORC2 is recruited to ribosomes and to phosphorylate the newly synthesized Akt at Thr450. Thr450 phosphorylation stabilizes the Akt protein by preventing K48-ubiquitination-mediated degradation. When cells receive signals from growth factors, a small fraction of resting Akt is further phosphorylated at Ser473 by mTORC2 and at Thr308 by PDK1, thus becoming fully activated. Active Akt not only controls its own substrates but also phosphorylates Sin1 at Thr86 to augment the activity of mTORC2, resulting in a positive feedback loop for more mTORC2 substrate phosphorylation. Alternatively, Sin1 could be phosphorylated at both Thr86 and Thr398, leading to dissociation of mTORC2 to negatively regulate the mTORC2–Akt axis. Finally, Akt Ser473 phosphorylation also primes Akt for the Lys48-linked polyubiquitination, leading to its degradation, thus preventing over-activation of the PI3K–Akt pathway.

Remarkably, the Akt Thr450 residue is highly conserved not only across species but also exists in many AGC kinases including PKC and PKA [85,86]. Although phosphorylation of this site was identified in the middle 90’s of the last century [92] as discussed above long before the function of mTORC2 was studied, the importance and implication of this site phosphorylation was not appreciated until the discovery of Sin1-mediated mTOR function [85,86]. Its major function is to regulate the stability of conventional (c)PKC and Akt [72,85,86,95]. When cellular nutrients are sufficient, mTORC1 stimulates Akt protein synthesis. During the translation process, mTORC2 is recruited to active ribosome and directly binds ribosomal protein L23 at the exit tunnel of the ribosomal complex [95] where mTORC2 phosphorylates newly synthesized Akt Thr450 at the turn motif (TM) but not Ser473 in the hydrophobic motif (HM) [95] (Fig. 3). This phosphorylation is critical for correct protein folding and stabilization of Akt by preventing co-translational ubiquitination and degradation [85,86] (Fig. 3). Interestingly, in Sin1- or mTORC2-deficient cells, the partially folded Akt or cPKC was protected by the molecular chaperone HSP90, thus maintaining a similar half-life in cells as that in wild type cells [85,86]. Consistently, dual inhibition of Sin1–mTORC2 and the chaperone HSP90 pathway synergistically suppressed the growth of leukemia tumor cells [96]. In response to growth factors or other extracellular cues, a small fraction of quiescent Akt is further phosphorylated at Ser473 and Thr308 to become fully activated. Active Akt not only controls its well-characterized substrates but also phosphorylates Sin1 at Thr86. Some believe that this phosphorylation leads to the augmentation of mTORC2 activity, resulting in a positive feedback loop [72,97,98] (Fig. 3), while others have shown evidence that phosphorylated Thr86 causes disruption of the mTORC2 complex thus suppressing tumor cell growth [17] (Fig. 3). We speculate that under different cellular environments, the same modification of Sin1 may lead to different outcomes. Furthermore, the Akt Ser473 phosphorylation primes Akt for Lys-48-linked polyubiquitination, leading to its degradation [99] (Fig. 3). This Sin1–mTORC2-mediated down-regulation of active Akt is likely a crucial step to prevent overt activation of the PI3K–Akt pathway, which has been linked to numerous human diseases [2,17].

**Figure 4.**
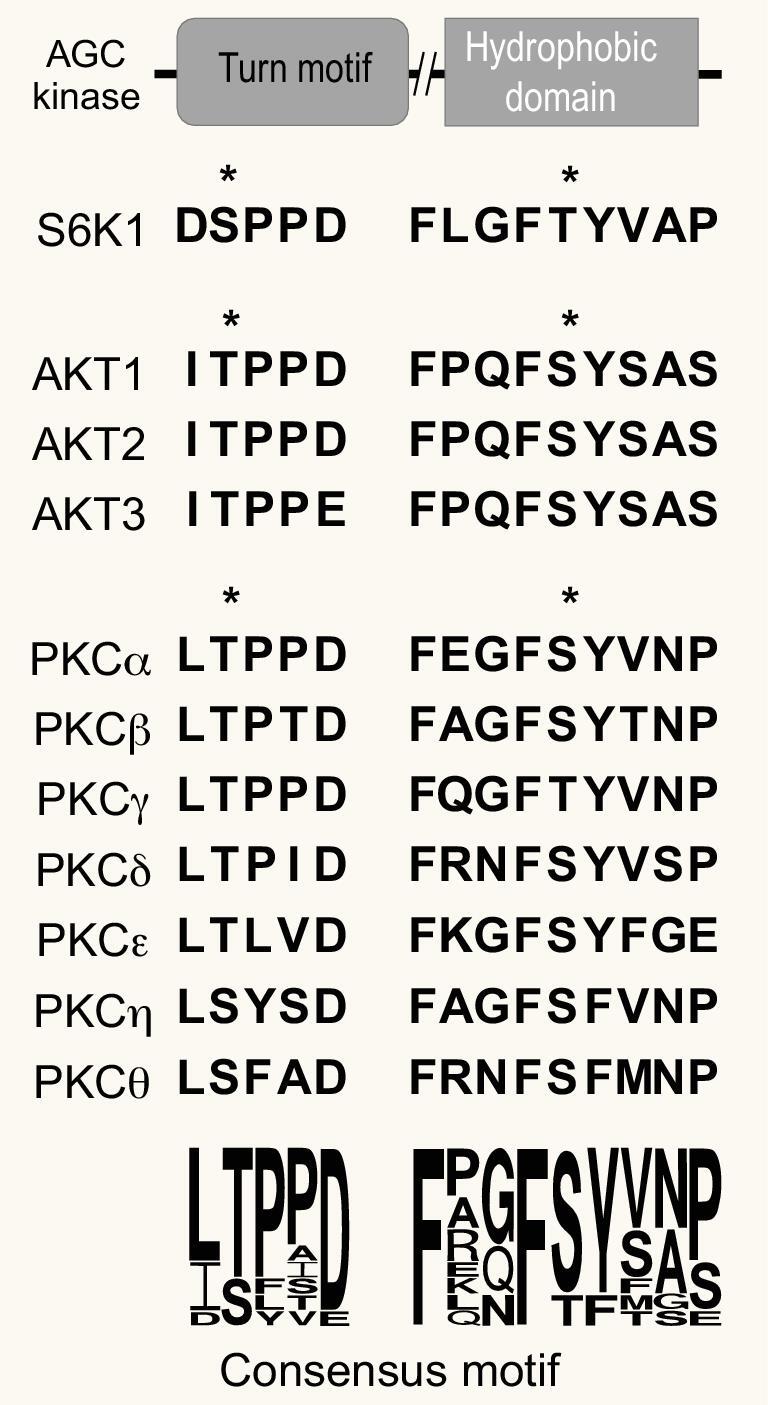
Conservation of the turn motif (TM) and hydrophobic motif (HM) of members of the AGC kinase family, and sequence alignment of the TM and HM domains of Akt and PKC. All the evolutionarily conserved sites that can be phosphorylated by mTORC2 are indicated by asterisks. The degree of the conservation of the consensus residues in the TM and HM domains is illustrated by the size of the specified residues at the bottom. All sequences are based on human sequences downloaded from Uniprot.

The TORC2–AGC kinase signaling axis is evolutionarily conserved. Both *S. cerevisiae* and *S. pombe* have Akt orthologues (YPK and Gad8, respectively) that can be phosphorylated by TORC2. However, their protein sequences are very different from mammalian Akt. In addition, Thr450 in the TM is also conserved in other AGC kinases like cPKC (Fig. 4). Therefore we speculate that besides Akt, this Sin1–mTOR-regulated kinase activation mechanism may also affect other kinases downstream of mTOR by regulating their stability and activity through the TM and HM sites.

## PHYSIOLOGICAL FUNCTION OF SIN1

### Sin1 function in immune responses

Sin1 plays a major role in the immune system, ranging from the development of immune cells, production of cytokines and generation of specific microenvironment [69,70,90,100]. Sin1 was first shown to regulate the number of Foxp3^+^ natural T regulatory cells [101]. A recent study also demonstrates that Sin1 is involved in the metabolism and proliferation of DN T cells in the thymus [69]. Sin1/mTORC2 controls the proliferation and development of DN stage T cells by regulating glycolytic metabolism through an Akt–PPARγ–PKM2 axis [69]. Moreover, Sin1 controls the homing of naïve T cells. Under normal state, Sin1 suppresses CXCR4 expression, and Sin1 deletion leads to an increased accumulation of naïve T cells in bone marrow [102].

Likewise, Sin1 is required for B cell development, antibody generation and humoral immunity response upon viral infection [70,100,103]. Sin1 plays a vital role in transducing BCR-mediated PI3K signals to Akt and regulates the stability of c-Myc and the activity of mTORC1 via GSK3 and TSC1/2, respectively [100]. This partly explains why in Sin1-null B cells there is a limited expansion of the B cell pool [70]. HSCs with compromised Sin1 also generate fewer immature B cells than wild type, due to higher expression levels of *il7r* [104]. Sin1 deficiency promotes *il7r* and RAG gene expression, pro-B cell survival and V(D)J recombination when cultured with IL-7. Although increased pro-B cell survival is observed, it is accompanied by decreased levels of IgM^+^ immature cells, indicating stagnation of B cell development [100].

Although no apparent defects in neutrophil development is observed in Sin1-deficient mice [105], Sin1 may regulate the activity of platelets [106]. Sin1 was found highly phosphorylated at Thr86 in platelets from patients with ST-elevation myocardial infarction (STEMI), accompanied by elevated mTORC2 activation and Akt Ser473 phosphorylation [106]. Sin1 deficiency in platelets attenuates the microthrombosis after ischemic conditions. The capability of Sin1 to reduce ROS levels under hypoxic conditions may prevent platelet activation and embolization in ischemic cardiovascular diseases [106]. Mechanistically, Sin1 may mediate the αIIbβ3-initiated outside-in signaling.

Apart from mediating the development of immune cells, Sin1 also contributes to the formation of a specific microenvironment in bone marrow as knockdown of Sin1 at mRNA level reduces low-dose irradiation-induced Akt Ser473 phosphorylation and subsequent responses in mouse osteoblasts, which inhibit osteoblast differentiation [107]. Sin1 may also regulate multiple cytokine-induced pathways since dysregulation of Sin1 led to decreased STAT1 and suppressed the transcription of several IFN-γ-induced genes [91]. As discussed above, Sin1 could also regulate type I IFN production and its antitumor responses [89].

It has been shown that abnormal PKCδ, which forms an overly stable interaction with Sin1, may contribute to SHORT syndrome [75]. CCDC28B, a protein associated with the Bardet–Biedl syndrome, is shown to interact with Sin1 and disrupt cilia formation, which is linked with proper signal transduction and immune responses [108]. Nijmegen breakage syndrome (NBS) protein NBS1 interacts with Sin1/mTORC2 [109]. Reduced Sin1 activity may prevent chronic rejection in allograft models [96,110].

Other functions of mTORC2 may include the regulation of the generation of surface receptors [111], the lineage differentiation of T cell subsets [112–116], the development of memory T cells [117], the development and activation of other immune cells like macrophages, dendritic cells and NK cells [118–120], the production of cytokines [121], the long-term survival of immune cells [122] and so on. However, the precise role of Sin1 in these processes requires more thorough studies with specific cellular and mouse models.

### Sin1/mTORC2 function in metabolism

mTOR is a well-studied key regulator of metabolism via integrating upstream signals from nutrients and growth factors upstream of many AGC kinases that subsequently act on a wide range of transcription factors, which controls the level of rate-limiting enzymes in the metabolic pathways [2,21,123,124]. AGC kinases can directly activate rate-limiting enzymes of metabolic pathways [125–127]. The deficiency of Sin1 is therefore expected to cause great changes in metabolism.

Changes in cell metabolism are tightly associated with the growth, activation and differentiation of many immune cells including CD4^+^ T cells [118]. Studies in cell lines have uncovered the function of Sin1 in metabolism regulation. Sin1/mTORC2 could regulate mSREBP1 levels, and knockout of Sin1 decreased mSREBP1 levels and its target genes including acetyl-CoA carboxylase (ACC) and fatty acid synthase (FASN), resulting in suppressed lipogenesis in cells [128]. It is also found that Sin1/mTORC2 was required for the hexosamine biosynthesis pathway (HBP), via glutamine: fructose-6-phosphate amidotransferase 1 (GFAT1) [129]. Sin1 also controls cell metabolism in response to extrinsic and intrinsic immune signals. Recent studies show that Sin1 is required for upregulation of PKM2 for glycolysis [69] and is crucial for proper B cell glycolysis and mitochondrial respiration under both resting and anti-IgM-stimulated conditions [70,130].

### Sin1 function in cancer

Dysregulation in the mTORC2 pathway caused by overexpression or upregulation of Sin1 is associated with many types of cancer [21,131]. For example, Sin1 is upregulated in primary breast cancer tissues. Its overexpression is associated with higher proliferation and metastasis of tumor cells [132]. Sin1 is also overexpressed in clinically aggressive thyroid cancer types, including medullary thyroid carcinomas and aggressive variants of papillary thyroid carcinoma, accompanied by strong activation of Akt [133]. In the above cases, dysregulation of Sin1 was usually associated with over-activated Akt and high c-Myc levels, which is likely the reason for augmented tumor growth. Moreover, Sin1 may promote the metastasis and epithelial mesenchymal transition (EMT) of tumor cells. In NSCLC tissues, MLL expression is upregulated, which may activate Sin1 epigenetically to promote EMT and proliferation of NSCLC [134]. Elevated Sin1 level is also associated with higher invasion and metastasis of HCC cells and may facilitate the development of HCC [135]. Though the specific mechanism still remains to be discovered, it is reasonable to predict that drugs targeting the overly activated mTOR pathway, especially mTORC2, would have a clinical significance in cancer treatment. Indeed, suppression of mTORC2 by expressing the truncated form of Sin1 significantly impairs tumor growth in the xenograft mouse model [136]. Similarly, a tumor suppressor Pdcd4 that could attenuate Sin1 translation was shown to prevent invasion of colon carcinoma [137] and a synthesized Rictor inhibitor, also targeting mTORC2 activity, could inhibit glioblastoma growth in a xenograft model [138]. Better understanding of the Sin1/mTORC2 signaling pathway will provide more clues to develop efficient cancer diagnosis and treatment methods.

### Sin1 function in neurons

TPKC, an important regulator of cytoskeletons, is directly regulated by Sin1/mTORC2, and its substrates include many important proteins in actin cytoskeletal rearrangement *in vivo*, including, but not limited to, GAP43, MARCKS and adducin [139]. In *Drosophila*, long-term memory was restored in aged flies and enhanced in young flies by direct activation of dTORC2. At the same time, actin polymerization in neurons was observed [140]. Similarly, deficiency of Rictor in mice reduced the level of actin polymerization, which is associated with impaired long-term memory and hippocampal potential [141]. Interestingly, short-term memory is not affected [142]. In humans, mTORC2 dysfunction is associated with irregular insulin/PI3K/Akt function, which is an important feature of Alzheimer's disease pathogenesis [143].

## PERSPECTIVE AND FUTURE STUDIES OF SIN1 REGULATION AND FUNCTION

### Targeting Sin1 as an mTORC2-specific inhibitor

Sin1, since its first discovery as an essential mTORC2 subunit in 2006, has been shown to have more and more indispensable functions in cell growth, metabolism, cancer and immunity. Given its potent roles in mTORC2 assembly, regulation and substrate specificity in immune function and cancer development, it is natural to believe that approaches targeting the Sin1–mTORC2 interaction may yield fruitful outcomes with a clinical impact. Considering the lack of mTORC2-specific inhibitors at the moment, it is likely that Sin1 could be an ideal target for mTORC2 inhibitors.

Although much has been learned about the structure and function of Sin1, more questions remain to be answered. For example, why does Sin1 have multiple isoforms and what are their physiological functions? What controls mTORC2 subcellular localization? How would mTORC2 signaling crosstalk with that of mTORC1, and how would it impact cellular metabolic re-programing? In addition, it is already known that Sin1 and mTOR subcellular localizations are not entirely overlapping, indicating that Sin1 has mTOR-independent functions. Consistently, although both Sin1 and Rictor are required for mTORC2 integrity and functions [10], deletion of Sin1 and Rictor individually shows different phenotypes in neutrophils [105], in T cells [101,111] and in other cell types [103]. These data strongly suggest that Sin1 may have distinct functions besides mTORC2.

### Sin1 mediates the crosstalk between mTORC1 and mTORC2

It is well appreciated that the main function of mTORC1 is sensing the intracellular nutrient levels, and when adequate, directs the biosynthesis of macromolecules such as proteins and nucleic acids as building blocks for cell growth and proliferation. On the other hand, mTORC2 may not be directly involved in the biosynthesis of such macromolecules but instead, it senses the extracellular or intracellular conditions for cells to determine if it is suitable for starting a biosynthesis process. For instance, if there are abundant levels of growth factors, mTORC2 may send a signal to cells for rapid biosynthesis of macromolecules. In contrast, if cellular stresses are sensed by mTORC2, it may signal to the cells to stop biosynthesis of macromolecules, thus saving energy that would be needed to respond to the adverse situation. Therefore, one outstanding question in the field is how the two distinct mTOR complexes, mTORC1 and mTORC2, coordinate the nutrient- and growth factor- or cellular stresses-mediated signals in controlling cell growth and metabolism. Since the two mTOR complexes share key components, which have evolved to regulate similar cellular growth and metabolism processes, we believe that they should be involved in sensing nutrients and cellular signals synergistically to regulate those key cellular functions (Fig. 1).

Previous studies have provided strong evidence showing that mTORC2 could send positive signals via the AGC kinase Akt to inhibit the TSC complex, which is a negative regulator of mTORC1, thus augmenting the mTORC1 activity and promoting optimal cell growth [4,144,145]. Since this cross-regulation of mTORC1 by mTORC2 is mediated directly through a small GTPase RheB, it is interesting to know if mTORC2 could also regulate mTORC1 by targeting its upstream nutrient-sensing components [12,146]. Multi-protein complexes such as Gator1, Gator2 and Kicstor etc. have been identified in the past several years acting upstream of mTORC1 with important roles in sensing nutrients such as glucose, amino acids and cellular energy levels [12,147–150]. Given that most of those studies focus only on the roles of those complexes in response to nutrients, little is known at the moment how signals activating through mTORC2 may be linked to these nutrient-sensing components of mTORC1. In our study, we found that mTORC2 deficiency could lead to increased mTORC1 activity under certain conditions [10,85] (data not shown), while over-activation of mTORC1 also restricts mTORC2 activity, either through the mTORC1-mediated phosphorylation and activation of Grb10, a negative regulator of insulin/IGF-1 receptor [12,151], or by S6K1 phosphorylation and degradation of insulin receptor substrate 1 (IRS1) to suppress mTORC2 activity [152]. Mathematical modeling of phospho-proteomic data in insulin-treated adipocytes revealed a network consisting of both a negative signal from S6K to Rictor/mTORC2 and a positive signal from Akt to Sin1/mTORC2 [97,98]. This model also predicts that under insulin-resistant diabetic conditions, mTORC1 and mTORC2 crosstalk to each other to enhance the positive feedback between mTORC2 and Akt, whereas the negative signal from S6K is reduced, resulting in increased overall Akt-Ser473 phosphorylation and likely Akt activity in diabetic patients [152]. Consistently, the computational simulation model that used constitutively active Akt with Ser473 phosphorylation in type 2 diabetes patients showed decreased mTORC1 activity [153]. It was also revealed that S6K could phosphorylate Rictor at Thr1135 to suppress mTORC2 formation [98]. Furthermore, it was found that Foxo1/3, a well-characterized downstream target of mTORC2, may activate Sestrin3 to decrease mTORC1 activity [154].

S6K may also phosphorylate Sin1 at Thr86 and Thr398, which was proposed to dissociate Sin1 from mTORC2, leading to a decreased mTORC2 activity [17] (Fig. 3). This negative feedback model seems to be supported by the fact that a Sin1-R81T mutation was associated with ovarian cancer [17]. This mutation results in a deficiency of Sin1 phosphorylation at Thr86 and sustained activation of mTORC2 and Akt [17]. Interestingly, it was also shown in this study by Liu *et al.* [17] and from an independent study [97] that Akt is also a kinase for Sin1 Thr86 phosphorylation. In this case, it was Akt that mediated Sin1 Thr86 phosphorylation to positively regulate mTORC2 activity [98], and a computational simulation also supports the positive regulation model [153]. Recent data from our group with Sin1 Thr86 phosphorylation-deficient knockin mice support Sin1 Thr86 phosphorylation as a positive regulator of mTORC2 (data not shown).

While mTORC1 and mTORC2 are thought to be activated by different upstream cues, increasing evidence suggests that a wide spectrum of environmental and intrinsic cues can activate both complexes at the same time or even synergistically. For instance, Ras homolog enriched in striatum [155], a small G protein, has been shown to interact with both complexes and activate mTOR activity [155]. DEPTOR, capable of binding both mTOR complexes, could suppress the formation of both complexes [156]. On the other hand, small GTPase Rac1 could positively regulate both mTOR complexes [157]. Activation of both mTORC1 and mTORC2 could lead to upregulation of protein translation via the mTORC1-mediated S6K activation [158] and the mTORC2-mediated Akt activation [159]. These data strongly indicate a common shared function of mTOR at the early stage of evolution. In this regard, phylogenetic studies of most components of the TOR pathway found that it originated before the Last Eukaryotic Common Ancestor [160]. mTORC1 and mTORC2 may result from an ancient genome duplication event [160]. Interestingly, in budding yeast, TOR2 is a subunit for both TORC1 and TORC2, suggesting that at this evolutionary stage the two complexes may have redundant function. Afterward, the two complexes diverged and evolved as separated modules to have different functions in different cellular processes [160].

Although most studies on mTORC2-mediated Akt phosphorylation and regulation focus on the HM site phosphorylation, it is still puzzling about the role of mTORC2 in the phosphorylation of the TM sites of AGC kinases such as Akt and cPKC that we identified a few years ago [10,85,86]. We showed that this highly conserved function of Sin1-mTORC2 is required for stabilizing the newly synthesized AGC kinases Akt and cPKC primarily through facilitating the proper folding of the nascent peptides. Since the phosphorylation of the Akt HM site is dependent on growth factor while that of the TM site seems not to be, we speculate that the phosphorylation of TM appeared earlier during evolution.

Whether it is a general form of regulation of the stability of many other nascent cellular proteins remains elusive. Considering that mTOR is a well-conserved protein kinase for protein synthesis, it is reasonable to believe that these two protein complexes should coordinately control the overall protein synthesis, which includes not only the polypeptide chain elongation, but also the quality of the nascent polypeptides. In this regard, to fully ensure that a newly synthesized protein functions properly, it is required to be properly folded, modified and transported to its proper location for function. In case any of those steps fail, the polypeptide should be degraded and recycled to prevent the accumulation of waste that is known to be the cause of many diseases [161,162]. We believe that mTORC2, which may not be directly involved in polypeptide enlongation, may serve this quality check and recycle function of the newly synthesized polypeptides. Consistently, this function of mTORC2 does not require growth factors or other extracellular stimuli, and could function constitutively [10,85].

**Figure 5.**
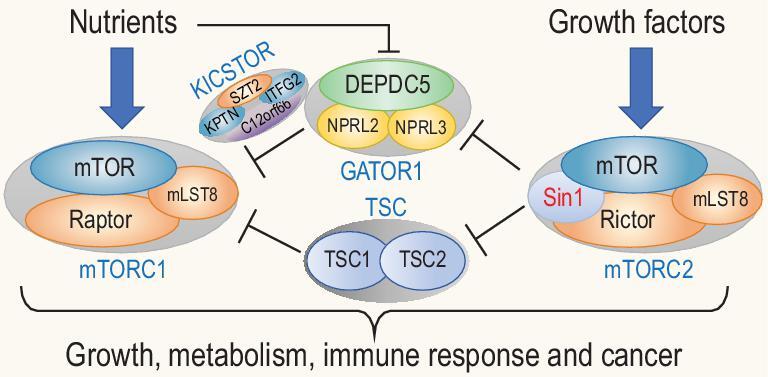
The newly proposed model for the crosstalk between mTORC1 and mTORC2 in regulation of cell growth, metabolism, immunity and cancer. In response to nutrients and growth factors, mTORC1 and mTORC2 phosphorylate their respective substrates as indicated to control major cellular functions as indicated. As illustrated, mTORC2 sends positive signals via Akt to mTORC1 by inhibiting its negative regulator TSC complex. Similarly, mTORC2 may also regulate mTORC1 activity by controlling the activity of its upstream nutrient-sensing components such as Gator1.

Finally, the precise molecular mechanism by which the mTORC1 and mTORC2 coordinately control protein synthesis and metabolism remains largely unknown. In search for such a crosstalk between these two complexes, we have recently identified components of Gator1 that could be regulated by Sin1, most likely via the mTORC2 function (data not shown and Fig. 5). Combined with the well-established mTORC2–Akt–TSC axis in mTORC1 regulation, we propose a new model showing that Sin1–mTORC2 may also directly regulate the Gator1–Kicstor complex to impact the mTORC1 activity, and together to control cell growth, metabolism, immune responses, and perhaps even cancer development. Future investigation of this area is likely to yield fruitful results and may lead to the identification of new therapeutic targets for the treatment of various diseases due to abnormal metabolic regulation.
